# Research on the correlation of immunity in patients with chronic insomnia

**DOI:** 10.3389/fpsyt.2022.1034405

**Published:** 2022-10-18

**Authors:** Li Nie, Xian-li Pan, Xiao-bao Zhang, Shan Zhang, Ji-Xian Rao, Zeng-feng Su

**Affiliations:** Department of General Medicine, Chaohu Hospital Affiliated with Anhui Medical University, Chaohu, China

**Keywords:** insomnia, immunity, lymphocyte subsets, immunoglobulin, infection, platelet-to-lymphocyte ratio, neutrophil-to-lymphocyte ratio

## Abstract

**Purpose:**

To investigate the changes in immunity and clinical infection events among patients with chronic insomnia.

**Materials and methods:**

Forty-two patients with chronic insomnia (age = 64.44 ± 10.53) and 47 normal controls (age = 67.08 ± 7.822) were selected to determine differences in data, such as complete blood counts (CBCs), biochemical indices, lymphocyte subsets, immunoglobulin (Ig), complement C3 and C4 and interleukin-6 (IL-6), as well as to compare the incidence of clinical infection between the two groups.

**Results:**

There were significant differences in erythrocyte, hemoglobin, hematocrit, albumin, globulin, creatinine, IgG, IgG/IgM ratio, CD4^+^ T-lymphocytes, CD19-lymphocytes, CD4^+^/CD8^+^ ratio, platelet/lymphocyte ratio, CD19/CD3 ratio, and clinical infection events between the chronic insomnia group and the control group (*p* < 0.05). There was no significant difference in neutrophil, lymphocyte, monocyte, and platelet counts; lymphocyte subsets CD8^+^ T and CD56^+^; platelet-to-lymphocyte ratio (PLR); neutrophil-to-lymphocyte ratio (NLR); complement C3; complement C4; IgM; IgA; and IL-6 between the experimental group and their controls (*p* > 0.05). The systolic and diastolic blood pressures of the chronic insomnia group did not vary widely from those of the controls (*p* > 0.05).

**Conclusion:**

Patients with chronic insomnia have immunological abnormalities, characterized by a higher incidence of clinical infection.

## Introduction

By definition, chronic insomnia is used to describe any illness characterized by having trouble falling or staying asleep, waking up earlier than desired, keeping an irregular bedtime, and failing to sleep without the company of parents or others, lasting more than 3 nights a week for 3 months (1). The global epidemiological statistics of insomnia range from 10 to 48% (2, 3); the incidence of insomnia among females is 1.5 times that among males (4); and the incidence of chronic insomnia is gradually increasing in young, middle-aged and elderly people (5, 6). The prevalence of insomnia among the elderly is as high as 60.9% (7). The median age of Chinese insomnia patients was 43.7 (8). Considering that the prevalence of insomnia increases with age, our study selected middle-aged and elderly insomnia patients over 45 years old.

It is well known that the relationship between sleep and immunity is bidirectional. Inflammation caused by microbial infections that activate the immune system can lead to fatigue and increased sleep desire. Good sleep feeds back to the immune system, helping the host defend him-/herself to reduce the body's inflammatory response. Sleep has been physiologically considered a restorative or recovery phase (9). There is an inextricable connection between sleep and immunity, in which the activation of the immune system could affect sleep quality, whereas the latter in turn may contribute to the former (10).

Many studies have shown that chronic insomnia can increase the incidence of cardiovascular diseases (11–14), mental diseases (15, 16), digestive system diseases (17–20), autoimmune rheumatic diseases (21–24), metabolic syndrome, inflammation, and cancer. The field bridging insomnia and appeal disease has been defined as neuroimmunology (25–28).

Studies have shown that chronic insomnia is related to neutrophils, lymphocyte subsets (29), cytokines (30), immunoglobulins and complement (31). However, their relationship is still controversial, and there are few studies on the correlation between chronic insomnia and routine blood tests, platelet/lymphocyte ratio, neutrophil/lymphocyte ratio, and clinical infection events.

To clarify the changes in immunity in patients with chronic insomnia, this article sheds light on the correlation between chronic insomnia and immune function through the detection of CBC, biochemical indicators, lymphocyte subsets, immunoglobulin, complement C3, complement C4, and interleukin-6.

## Materials and methods

### Research subjects

Forty-two patients with chronic insomnia who visited the Department of General Medicine and Department of Sleep Disorders, Chaohu Hospital Affiliated with Anhui Medical University, from November 2021 to June 2022 were included. Forty-seven healthy subjects admitted to the Physical Examination Center of Chaohu Hospital affiliated with Anhui Medical University at the same time served as the control group. All subjects signed informed consent forms. The inclusion criteria were the following: (1) The disease duration was at least 3 months; (2) Age ≥ 45 years old; (3) Education Level: Junior high school or above, with no impediment in comprehension; (4) Pittsburgh Sleep Quality Index (PSQI) > 7; and (5) The total score of Hamilton Depression Rating Scale (HAMD-17) <17. The exclusion criteria were the following: (1) consumption of antidepressants, antipsychotics, and hypnotics within 2 weeks; (2) strong repulsion for inspection, unable to complete tests; (3) presence of neurocognitive disorders; (4) complications of other mental illnesses; (5) pregnant or lactating; (6) severe heart, liver, kidney or endocrine system diseases; (7) suffering from malignant tumors, blood diseases or autoimmune diseases; (8) suffering from infectious diseases (including viral and bacterial infection) within nearly half a month; and (9) sleep disorders other than insomnia as assessed by screening polysomnography [PSG, including an apnea–hypopnea index (AHI) of ≥5 episodes per hour and periodic limb movement arousal index (PLMI) of ≥5 episodes per hour].

This was a cross-sectional study that was reviewed and approved by the Ethics Committee of Chaohu Hospital affiliated with Anhui Medical University. All subjects signed informed consent forms.

### Research methods

General demographic information included gender, age, nationality, and educational level; the assessment criteria for sleep were the PSQI and sleep diary; and the assessment criteria for depression was the Hamilton Depression Rating Scale (HAMD-17) to assess underlying conditions.

#### Immunological indices

Blood cell count and the percentage and count of lymphocyte subsets detected by flow cytometry; and IgG, IgA, IgM and complement C3 and C4 assessed by immunoturbidimetry. Interleukin-6 (IL-6) was determined by the chemiluminescent immunoassay. Blood samples were collected from each subject between 7:30 a.m. and 8:30 a.m., with follow-up testing completed by 10:00 a.m. “Clinical infection events” were assessed through questionnaires, which assessed whether the respondents had experienced the following conditions in the past 3 months: sore throat, cough, upper respiratory tract infection, cold, oral ulcer, periodontitis, herpes zoster, acute gastroenteritis and urinary tract infection.

### Statistical methods

The statistical software used was SPSS 26.0, and the Shapiro–Wilk test was performed to assess normality (measurement data conforming to a normal distribution are expressed by x ± s). The chi-square test was used to analyze differences between groups of categorical variables. A t test was used for intergroup comparisons of normally distributed data, while the Mann–Whitney U test was used to analyze non-normally distributed variables. Point-biserial correlation analysis was performed to assess the correlation between chronic insomnia and each index. Binary logistic regression was employed to analyze the relationship between chronic insomnia and clinical infection events and sex. If *p* < 0.05, the differences were considered statistically significant.

## Results

### Comparison of general data

There were no significant differences in sex, age composition ratio, blood pressure or history of chronic diseases (hypertension, type 2 diabetes mellitus, hyperlipidemia) between the two groups (*p* > 0.05). Chronic insomnia patients had a lower total sleep time (TST), REM sleep, and sleep efficiency, while NREM sleep and WASO increased. In summary, the objective sleep time and sleep quality of chronic insomnia patients in this study were shorter. See Table 1 for more details.

**Table 1:** Comparison of general data between the two groups.

**Variables**	**Insomnia group (*n* = 42)**	**Control group (*n* = 47)**	**x^2^/*t* value**	***P*-value**
Male/Female	22/20	24/23	0.015	0.901
Hypertension (Yes/No)	14/28	18/29	0.218	0.641
Diabetes mellitus type 2 (Yes/No)	7/35	7/40	0.049	0.826
Hyperlipidemia (Yes/No)	14/28	16/31	0.005	0.946
Age	64.44 ± 10.53	67.08 ± 7.82	−1.217	0.228
Systolic blood pressure (mmHg)	137.54 ± 25.12	135.98 ± 21.96	0.791	0.992
Diastolic blood pressure (mmHg)	78.72 ± 11.39	80.25 ± 12.27	0.656	0.61
Polysomnogram (*n* = 30)		
Total sleep time (min)	297.57 ± 41.74	
Sleep efficiency (%)	73.98 ± 4.2	
Time in wake after sleep onset (min)	105.73 ± 27.03	
NREM sleep stage 1 (%)	16.00 ± 6.00	
NREM sleep stage 2 (%)	58.77 ± 12.64	
NREM sleep stage 3 (%)	12.08 ± 4.49	
REM sleep (%)	13.15 ± 5.96	

### Comparison of CBC and biochemical indices

The chronic insomnia group apparently varied from the controls in terms of erythrocyte, hemoglobin, hematocrit, total protein, albumin, globulin, creatinine, platelet-to-lymphocyte ratio (PLR), and neutrophil-to-lymphocyte ratio (NLR) (*p* < 0.05), while showing no obvious difference (*p* > 0.05) in white blood count (WBC), neutrophil count, monocyte count, eosinophil count, basophil count, platelet count, reticulocyte count, ALT, AST, AST-to-ALT ratio, GGT, total bilirubin, direct bilirubin, indirect bilirubin, prealbumin, urea, glucose, total cholesterol, triglyceride, high-density lipoprotein, and low-density lipoprotein. See Table 2 for more details.

**Table 2:** Comparison of CBC and biochemical indices between the two groups.

**Variables**	**Chronic insomnia group**	**Control group**	***P*-value**	**x^2^/t value**
WBC	6.363 ± 2.244	6.058 ± 1.262	0.475	0.720
Neutrophil count	3.972 ± 1.992	3.5644 ± 1.012	0.275	1.105
Monocyte count	0.378 ± 0.151	0.354 ± 0.090	0.807	0.423
Eosinophil count	0.149 ± 0.147	0.152 ± 0.138	0.904	−0.121
Basophil count	0.037 ± 0.027	0.035 ± 0.029	0.721	0.359
Platelet count	187.57 ± 58.73	164.62 ± 47.446	0.057	1.933
Reticulocyte count	0.0761 ± 0.02	0.0807 ± 0.02	0.307	−1.029
ALT	22.65 ± 12.351	21.83 ± 7.669	0.734	0.342
AST	23.65 ± 6.989	24.39 ± 5.908	0.608	−0.515
AST-to-ALT ratio	1.192 ± 0.375	1.1795 ± 0.255	0.866	0.169
GGT	28.21 ± 29.666	26.07 ± 11.148	0.691	0.400
Total bilirubin	15.862 ± 5.886	17.413 ± 5.833	0.245	−1.171
Direct bilirubin	5.42 ± 2.009	5.320 ± 1.965	0.836	−0.208
Indirect bilirubin	11.15 ± 3.948	11.820 ± 4.158	0.488	−0.698
Albumin	251.7 ± 54.502	273.97 ± 48.13	0.081	−1.775
Urea	6.538 ± 1.991	11.578 ± 2.056	0.364	−0.914
Glucose	5.60 ± 1.350	5.643 ± 1.273	0.883	−0.148
Total cholesterol	4.702 ± 0.893	4.8234 ± 0.798	0.519	−0.647
Triglyceride	1.483 ± 0.602	1.6632 ± 0.833	0.259	−1.136
High-density lipoprotein	1.264 ± 0.307	1.215 ± 0.355	0.510	0.662
Low-density lipoprotein	2.630 ± 0.849	2.726 ± 0.727	0.586	−0.547
Erythrocyte	4.450 ± 0.417	4.677 ± 0.468	0.027	−2.257
Hemoglobin	134.49 ± 13.069	141.29 ± 16.347	0.048	−2.012
Hematocrit	40.991 ± 3.7341	43.104 ± 4.676	0.032	−2.185
Total protein	73.088 ± 3.767	76.267 ± 3.368	<0.001	−3.968
Albumin	46.02 ± 3.743	47.511 ± 1.725	0.033	−2.018
Globulin	26.983 ± 4.126	29.155 ± 3.245	0.018	−2.431
Creatinine	67.971 ± 19.445	78.193 ± 19.641	0.023	−2.311
Platelet-to-lymphocyte ratio	135.148 ± 42.834	86.438 ± 25.567	<0.001	5.953
Neutrophil-to-lymphocyte ratio	3.009 ± 2.214	1.946 ± 0.853	0.01	2.708

### The comparison of immunological indices

Patients with chronic insomnia significantly varied from their controls (*p* < 0.05) in terms of IgG, IgG/IgM ratio, CD4^+^ T-lymphocyte count/CD8^+^ T-lymphocyte count ratio, lymphocyte count, CD3 T lymphocytes, CD4 T lymphocytes, CD8 T lymphocytes, and NK lymphocytes (CD56), while showing no apparent difference in complement C3 and C4, immunoglobulin A and M, IL-6, B lymphocytes (CD19), IgG/IgA ratio, IgM/IgA ratio, or C3/C4 ratio (*p* > 0.05). See Table 3 for more details.

**Table 3:** Comparison of immunological indices between the two groups.

**Variables**	**Chronic insomnia group**	**Control group**	***P* value**	**x^2^/t value**
Complement C3	0.970 ± 0.152	0.925 ± 0.140	0.164	1.404
Complement C4	0.250 ± 0.061	0.235 ± 0.052	0.236	1.195
Immunoglobulin A	2.365 ± 0.853	2.713 ± 1.060	0.114	−1.598
Immunoglobulin G	12.296 ± 2.344	14.553 ± 2.277	<0.001	−4.355
Immunoglobulin M	1.126 ± 0.516	1.109 ± 0.879	0.916	0.106
IgG/IgM ratio	13.038 ± 6.224	17.216 ± 8.068	0.013	−2.547
IL-6	3.436 ± 3.039	3.800 ± 4.266	0.665	−0.435
CD4^+^ T-lymphocyte count/CD8^+^ T-lymphocyte count ratio	1.434 ± 0.70598	1.1337 ± 0.46735	0.043	*2.08*
Lymphocyte count	1,436.86 ± 362.636	1,957.23 ± 499.155	<0.001	*−5.223*
CD3 T lymphocytes	834.894 ± 221.787	1,132.11 ± 396.402	<0.001	*−3.936*
CD4 T lymphocytes	449.845 ± 126.125	560.479 ± 171.695	0.002	*−3.175*
CD8 T lymphocytes	365.298 ± 140.517	550.476 ± 263.237	<0.001	*−4.053*
NK lymphocytes (CD56)	387.621 ± 235.678	548.128 ± 307.722	0.013	*−2.539*
B lymphocytes (CD19)	148.523 ± 71.807	149.555 ± 75.456	0.951	*−0.062*

### Comparison of clinical infection prevalence

The prevalence of clinical infection in a person, such as respiratory system infection, urinary system infection, oral ulcer and herpes zoster, was “1” if it occurred in the last 3 months and “0” otherwise.

According to binary logistic regression, the incidence of clinical infection was significantly higher among patients with chronic insomnia than controls [*p* = 0.008, OR (95% CI) = 3.51 (1.382–8.915)].

### Point-biserial correlation analysis

The results suggest that chronic insomnia has a negative correlation with erythrocytes, hemoglobin, hematocrit, albumin, globulin, creatinine, IgG, IgG/IgM ratio, lymphocyte count, CD3 T lymphocytes, CD8 T lymphocyte count, CD4 T lymphocyte count and NK lymphocyte count, while maintaining a positive correlation with CD4^+^ T-lymphocyte count/CD8^+^ T-lymphocyte count ratio, neutrophil-to-lymphocyte ratio, platelet-to-lymphocyte ratio, and clinical infection events. See Table 4 for more details.

**Table 4:** Point-biserial correlation analysis.

**Variables**	***p***	***R***
Erythrocyte	0.027	−0.248
Hemoglobin	0.048	−0.222
Hematocrit	0.032	−0.240
Albumin	0.033	−0.259
Globulin	0.018	−0.287
Creatinine	0.023	−0.253
Immunoglobulin G	<0.001	−0.436
IgG/IgM ratio	0.013	−0.277
Lymphocyte count	<0.001	−0.504
CD3 T lymphocytes	<0.001	−0.407
CD8 T lymphocytes	<0.001	−0.389
CD4 T lymphocytes	0.002	−0.338
NK lymphocytes (CD16CD56)	0.013	−0.276
CD4^+^ T-lymphocyte count/ CD8^+^ T-lymphocyte count ratio	0.028	0.251
Neutrophil-to-lymphocyte ratio	0.004	0.318
Platelet-to-lymphocyte ratio	<0.001	0.582
Clinical infection events	<0.001	0.509

## Discussion

In today's fast-growing society, the prevalence of chronic insomnia continues to rise annually. Total sleep time, sleep efficiency, and percent REM sleep decrease significantly with age, while sleep latency, percent N1, percent N2, and WASO increase with age (32). The middle-aged and elderly patients with chronic insomnia selected in this study all had moderate to severe insomnia. The objective results showed that their total sleep time, REM sleep, and sleep efficiency decreased, and NREM sleep and WASO increased, which is consistent with the above research results. Although the pathogenesis of chronic insomnia has not yet been fully elucidated, some researchers have put forward neurobiological and psychological models, leading to growing attention on immune factors.

### CBC and biochemical changes

The results showed that patients with chronic insomnia tended to have lower erythrocyte, hemoglobin, hematocrit, albumin, and creatinine levels than normal controls while maintaining a higher neutrophil-to-lymphocyte ratio (NLR) and platelet-to-lymphocyte ratio (PLR).

There are few studies on the correlation between erythrocytes and hematocrit. Nevertheless, some studies suggest that the systemic oxidative stress of insomnia patients increases as the level of antioxidant enzymes decreases (33). In addition, oxidative stress can strengthen the sensitivity of erythrocytes to shear-mediated damage. Some studies even indicate that oxidative stress can cause chronic damage to erythrocytes and hemoglobin, thus leading to chronic anemia (34). Moreover, some research evidence has shown that sleep disorders and abridged sleep time (<5 h) can cause lower hemoglobin levels (35). Given the positive correlation between sleep time (<5 h) and hematocrit, a longer sleep time (>7.5 h) can result in higher hematocrit, which is more evident in men (36).

Most of the studies on the correlation of chronic insomnia and immunity involve sleep deprivation (SD) for confirmation, which includes partial sleep deprivation (PSD) and total sleep deprivation (TSD). After total sleep deprivation (TSD), we found a lower level of erythrocytes and hemoglobin in mice and more immature erythrocytes, which may result from decreased erythropoiesis after sleep deprivation (37).

There was an inverted U-shaped relationship between sleep time and albumin level, wherein albumin reaches its maximum when sleep time is 7.5 h and significantly decreases when sleep time is <5 h or more than 9 h (38). Albumin, the most abundant plasma protein produced by the liver, is also an important indicator reflecting the body's nutritional status. Some studies have shown that albumin injected into young rats can promote non-rapid eye movement (NREM) sleep, leading to higher sleep quality (39). Further academic evidence has justified that low albumin and hemoglobin levels can directly result in insomnia and poor sleep quality (40, 41). The results of this study are consistent with those reported in the literature. However, the reasons for the decrease in erythrocytes, hemoglobin, and hematocrit caused by chronic insomnia remain unclear and are assumed to be due to oxidative stress and poor nutritional status.

It is worth noting that this study revealed that NLR and PLR in the chronic insomnia group were higher than those in normal controls. NLR and PLR can reveal systemic inflammatory responses, which are conducive to an increase in neutrophils and platelets and a decrease in lymphocytes, making their ratios a valuable tool for indirect assessment of inflammatory status and cellular immunity (42) in a variety of diseases, such as malignancies (including hematological malignancies), respiratory infections, gastrointestinal diseases, cardiovascular and cerebrovascular diseases (such as acute coronary syndrome, cerebral hemorrhage), systemic diseases (diabetes mellitus, diabetic foot) and, most recently, COVID-19. Patients with the above illnesses showed higher PLR and/or NLP than normal controls (43–49). More research data have indicated that during the COVID-19 pandemic, hospitalized positive cases with sleep disorders presented lower absolute lymphocyte counts and higher NLRs than those without sleeping problems (50). The method of obtaining PLRs and NLRs is simple, fast and low-cost, and the increase in these markers is related to many diseases, which is of great significance to research on the correlation between chronic insomnia and other diseases.

### Lymphocyte typing and changes in IL-6

In this study, we found that the numbers of total lymphocytes, CD3 T lymphocytes, CD8 T lymphocytes, CD4 T lymphocytes, and NK lymphocytes (CD56, CD16) were significantly lower in patients with chronic insomnia than in controls. Although the former group showed a strikingly higher percentage of B lymphocytes (CD19) than the latter group, there was no significant difference in their counts compared with those of the controls.

If sleep deprivation lasts one night, lymphocytes (CD4, CD16, CD56, and CD57) will be inhibited; if it exceeds 48 h, DNA synthesis of lymphocytes will decrease, and phagocytic function will decline if the deprivation extends beyond 72 h (51). Both the number and activity of NK lymphocytes will decrease after partial sleep deprivation, whereas their activity could return to the baseline level after a night of good sleep (52). Lymphopenia, neutrophilia, and mononucleosis during sleep deprivation can result in mildly progressive leukocytosis (41). Further research has shown that chronic insomnia is significantly associated with a decrease in lymphocyte subsets (i.e., CD3^+^, CD4^+^, and CD8^+^) but is slightly attributed to a decreased total lymphocyte count (51). These research data are in accordance with the results of this study.

IL-6 is a cytokine produced by lymphocytes and non-lymphocytes. Under physiological conditions, the secretion of IL6 exhibits a circadian rhythm with a biphasic pattern featuring two nadirs at 8:00 a.m. and 9:00 p.m. and two peaks from 5:00 p.m. to 7:00 p.m. and from 4 a.m. to 5 a.m., wherein it reaches a zenith at 5 a.m. (25). Mean IL-6 levels were significantly higher in patients with insomnia during the presleep period in the afternoon and evening (from 5 p.m. to 11 p.m.) than in controls. In addition, a cosine analysis result suggested that compared with controls, the prominent peaks of IL-6 secretion in insomniacs varied significantly from morning (5 a.m.) to evening (8 p.m.) (53). However, the results of a recent article positing the absence of significant differences in the mean IL-6 secretion within 24 h between chronic insomnia patients and controls (54) are consistent with the results of this study. Of course, the reason for this result may be that we ignored the circadian rhythm changes caused by chronic insomnia.

### Changes in immunoglobulin and complement

Immunoglobulin (Ig) refers to a globulin that has the activity or chemical structure of an antibody (Ab) and is similar to an antibody molecule. It comprises two identical light chains and two identical heavy chains connected by interchain disulfide bonds to form a four-peptide chain structure. Immunoglobulins are divided into five classes, namely, IgG, IgA, IgM, IgD and IgE, and a remarkable alleviation in immunodeficiency diseases, such as HIV, chronic lymphocytic leukemia, and thrombocytopenia, has been seen after injection of synthetic immunoglobulin (55).

However, current studies on the correlation between chronic insomnia and immunoglobulin are still lacking, and most are even controversial. In our experiment, we witnessed the first increase in IgM within 3–5 days of sleep deprivation, followed by an obvious augmentation in IgG and circulating IgA on Day 5 or longer and a global rise in all immunoglobulin subtypes on Day 20 (37). Nevertheless, other studies have not been able to replicate this finding, one of which showed that IgG and IgM levels after sleep deprivation, although they showed an increasing trend, did not change significantly compared with those of people who slept well (31). Another study indicated that circulating IgA decreased after 4 days of selective REM sleep deprivation, despite no drop in IgG and IgM (56). We found that IgG and IgG/IgM in patients with chronic insomnia were significantly lower than those in normal controls.

Complement is a serum protein that can mediate immune and inflammatory responses after enzymatic activation, although it is destructive to many other autoimmune and inflammatory diseases once it is activated inappropriately (57). Relevant research has indicated that complement factors C3 and C5 increase after acute sleep deprivation (23). After experimental sleep deprivation, complements (C3, C5, C3a, C5a) and receptors (C3aR, C5aR) in the rat hippocampus increased (58). However, in other studies, researchers found no changes in complements C3 and C4 or receptors C3aR and C5aR after acute complete sleep deprivation or prolonged REM sleep deprivation (56, 59). There is sufficient evidence to justify the influential role of complement inhibitors in several diseases associated with gene dysregulation. Plasma C3a indicates complement activation that performs more fully during sleep than at night (59). We found that the levels of C3 and C4 in the patients with chronic insomnia were slightly higher than those in the control group, but the difference was not significant.

### Clinical infection events

As shown above, patients with chronic insomnia may be immunocompromised, enduring higher susceptibility to infectious diseases. Some academic results have shown that patients with sleep disorders are 1.23-fold more likely than normal controls to become infected with herpes zoster (60). Compared with those who sleep more than 7 h, those who get <5 h of sleep have a greater risk of contracting the common cold (61). Compared with those who sleep 7–9 h, the risk of urinary tract infection and upper respiratory tract infection increases in patients who sleep <7 h (62). Sleep deprivation can contribute to the risk of periodontitis, exacerbate oral ulceration, and delay healing (63, 64). These research data are consistent with the results of this study, which suggests that patients with chronic insomnia are 3.51-fold more likely to develop clinical infections than normal controls. However, this study cannot determine whether respiratory infections or oral ulcers are associated with chronic insomnia. However, we must admit that patients with chronic insomnia are more prone to infection events.

### Limitations

The current study had several limitations. First, blood samples were obtained from 7:30 to 8:30 a.m. in this study, ignoring the changes in the circadian rhythm of the immune system. Second, the study sample was middle-aged and elderly, and most factors affecting immune function were excluded in the study design, but we did not exclude chronic diseases such as hypertension, type 2 diabetes mellitus and hyperlipidemia, which may affect the final results. Third, our sample size was relatively small, which may have limited our ability to detect differences in our primary outcomes due to the low statistical power. Fourth, the questionnaire format of clinical infection events made it difficult to determine the association of specific infections with chronic insomnia. Finally, PSG tests were consented to and were conducted only in the patients with chronic insomnia enrolled in the study, which may have affected the results. Future research should improve the PSG examination of the normal control group and increase the collection of samples to understand the correlation between sleep phase, sleep time, sleep efficiency and the immune system in chronic insomnia.

## Conclusion

This study shows that compared with those who sleep well, patients with chronic insomnia have weaker immunity, featuring significant changes in lymphocytes, immunoglobulin and other immune-related indicators, as well as some fluctuations in CBC and biochemical function. Most of the indicators are the same as those found in previous research on the correlation between chronic insomnia and immunity. Nevertheless, this paper also contains some controversial indices, and certain contents have seldom been discussed before, especially the high incidence of clinical infection in the group of chronic insomnia patients with immunological abnormalities, an increasingly researched cohort.

## Data availability statement

The raw data supporting the conclusions of this article will be made available by the authors, without undue reservation.

## Ethics statement

The studies involving human participants were reviewed and approved by Ethics Committee of Chaohu Hospital Affiliated to Anhui Medical University. The patients/participants provided their written informed consent to participate in this study.

## Author contributions

Z-fS and LN designed the study. LN and X-lP contributed to the conception of the study and drafted the manuscript. X-bZ, SZ, and J-XR contributed to the data collection. LN, X-IP, X-bZ, SZ, and J-XR contributed to the data interpretation. Z-fS critically contributed to revising the intellectual content of the manuscript. All authors contributed to the article and approved the submitted version.

## Funding

This study was funded by the Anhui Medical University Scientific Research Fund Project (No. 2020xkj219).

## Conflict of interest

The authors declare that the research was conducted in the absence of any commercial or financial relationships that could be construed as a potential conflict of interest.

## Publisher's note

All claims expressed in this article are solely those of the authors and do not necessarily represent those of their affiliated organizations, or those of the publisher, the editors and the reviewers. Any product that may be evaluated in this article, or claim that may be made by its manufacturer, is not guaranteed or endorsed by the publisher.
